# Novel cycloviruses identified by mining human blood metagenomic data show close relationship to those from animals

**DOI:** 10.3389/fmicb.2024.1522416

**Published:** 2025-01-22

**Authors:** Yuanqing Wang, Xiaojie Jiang, Yuan Xi, Siqi Wei, Songyi Ning, Wen Zhang

**Affiliations:** ^1^Institute of Critical Care Medicine, The Affiliated People's Hospital, Jiangsu University, Zhenjiang, China; ^2^Affiliated Hospital of Xuzhou Medical University, Xuzhou, Jiangsu, China; ^3^Department of Clinical Laboratory, Wuxi Blood Center, Wuxi, Jiangsu, China; ^4^Department of Microbiology, School of Medicine, Jiangsu University, Zhenjiang, Jiangsu, China

**Keywords:** Circoviridae, cycloviruses, phylogenetic, viral metagenomics, blood

## Abstract

The family Circoviridae includes the genera Circovirus and Cyclovirus. Cycloviruses have been found in serum samples from chronic HBV, HCV, or HIV-infected individuals as well as asymptomatic blood donors. However, research on cycloviruses is relatively limited. We used viral metagenomics to mine, analyze, and visualize the human blood virome, successfully identifying three new genomes, each encoding Rep and Capsid proteins. These proteins are crucial for viral replication and host-cell interaction: the Rep protein is involved in initiating viral genome replication, while the Capsid protein plays a key role in the assembly of new virions and the virus's ability to interact with host immune systems. Distance matrix and phylogenetic analyses show that these cycloviruses share high sequence similarity with viruses found in both humans and animals across different regions of Africa. This finding not only confirms the presence of previously uncharacterized cycloviruses in human blood, but also provides insight into their potential role in host transmission and their ecological significance. Further research is needed to explore the functional roles of these cycloviruses in viral pathogenesis, particularly how they may influence host immunity and contribute to chronic infections. Additionally, studies investigating the host range and mechanisms of cross-species transmission will be essential to understanding the broader implications of cycloviruses in human and animal health.

## 1 Introduction

The family Circoviridae includes viruses with small, circular, single-stranded DNA (ssDNA) genomes, which are usually small and about 1.7 to 2.1 kilo base pairs (bp) in length. Members of the family are divided into two genera, Circovirus and Cyclovirus, which are distinguished on the basis of the location of the replication start relative to the coding region and the length of intergenic spacers (Rosario et al., 2017). The genome structure usually contains two major open reading frames (ORFs): ORF1 encodes the Rep protein, which is responsible for viral replication and assembly and contains the characteristic sequence motifs of proteins involved in rolling-circle replication (RCR); the Rep protein is the most conserved protein in the cycloviruses; and ORF2 encodes the Capsid protein, which is the only structural protein and the major antigen of the viruses (Gainor et al., 2021). The Capsid protein plays a critical role in viral genome packaging, capsid assembly, and virus-host interactions (Zhan et al., 2020). Notably, the amino terminus of the Capsid proteins contains an arginine/basic amino acid-rich region, a feature that is closely related to their DNA-binding activity. In addition, the Capsid proteins exhibit large sequence differences between viruses (Gainor et al., 2021). Within each genus, the threshold for species delimitation is 80 % genome-wide nucleotide sequence identity (Breitbart et al., 2017). Members of the genus Circovirus have been identified only in vertebrates, whereas members of the genus Cyclovirus have been identified in both vertebrates and invertebrates (Rosario et al., 2012).

The genomic characteristics of Circoviridae confer a high mutation rate and variability, enhancing its capacity for inter-host transmission and adaptation. With the application of viral metagenomics, the presence of Circovirus has been elucidated in various animal hosts, including gorillas (Li et al., 2010), geese (Stenzel et al., 2018), dogs (Cardoso et al., 2023; Wang et al., 2021), and fish (Xi et al., 2023). It has been associated with canine gastroenteritis and diarrhea, respiratory diseases, and systemic vasculitis (Van Kruiningen et al., 2019; Thaiwong et al., 2016; Li et al., 2013). Additionally, it is linked to skin diseases in avian species (Franzo et al., 2022), as well as fatal conditions in wild boars and domestic pigs (Rudova et al., 2022). Recent studies have provided evidence of its presence in the livers of hepatitis patients and in the blood of drug users (Li et al., 2023; Pérot et al., 2023). Simultaneously, some researchers have reported the absence of circovirus in multiple blood samples (Pérot et al., 2024).

Cycloviruses have been implicated as potential contributors to gastrointestinal, respiratory, and neurological disorders in human patients (Phan et al., 2014, 2015; Smits et al., 2013). One specific strain of cyclovirus, known as CyCV-VN, was first isolated from cerebrospinal fluid samples of patients suffering from unexplained neurological disorders (Tan et al., 2013). Following this initial discovery, it has been identified in serum samples from individuals with chronic infections of HBV, HCV, or HIV in Italy (Macera et al., 2016). Furthermore, a high prevalence of CyCV-VN has been observed in plasma samples collected from asymptomatic blood donors in Madagascar (Sauvage et al., 2018).

Despite these findings, research on the clinical significance, pathogenic mechanisms, and host interactions of cyclovirus remains limited, underscores the need for further research. Preliminary evidence suggests that cyclovirus may be associated with hematological disorders and immune system abnormalities, but its full pathological potential remains unclear. This study aims to fill this critical gap by investigating the presence and genomic characteristics of cycloviruses in human blood. Specifically, we aim to identify novel cycloviruses, characterize their genetic diversity, and explore their potential role in human health, including their implications for viral transmission and pathogenicity.

## 2 Methods

### 2.1 Metagenome-assembled genomes

During the investigation of potential pathogenic viruses in humans, three usable libraries were downloaded from the SRA database (SRR16633776, SRR16633740, SRR16609930), submitted by Cordey et al. (2021) from the University Hospitals of Geneva. The sample processing methods have been described in previous publications (Cordey et al., 2021). SRA format files were converted to fastq format using Pfastq-dump v0.1.6 (https://github.com/inutano/pfastq-dump), and host sequences were removed using Bowtie2 v2.4.5 (Langmead and Salzberg, 2012; Langmead et al., 2019). Additionally, potential primer sequences in the raw reads were trimmed using Trim Galore v0.6.5 (https://www.bioinformatics.babraham.ac.uk/projects/trim_galore) and the subsequent files underwent quality control assessments employing parameter “–phred33 –length35 –stringency3 –fastqc.” Duplicate reads were marked using PRINSEQ-lite v0.20.4 (-derep 1) (Schmieder and Edwards, 2011), and the final library was assembled by an internal pipeline. Using default parameters, single-end reads were assembled with MEGAHIT v1.2.9 (Li et al., 2016). The resulting assemblies were subsequently imported into Geneious Prime v2022.0.1 for classification and manual confirmation (Kearse et al., 2012). Individual overlapping clusters serve as references for mapping to the original data using low sensitivity/fastest parameters in Geneious Prime. Additionally, mixed assembly was performed using MEGAHIT in conjunction with BWA v0.7.17 to search for potential low-abundance overlapping clusters from the unused reads (Buchfink et al., 2015).

### 2.2 Mining viruses

The blastx program within DIAMOND was utilized to compare alleles against the non-redundant protein database (nr). Based on the blastx program established in DIAMOND v2.0.15 (Buchfink et al., 2021), an E-value threshold of < 10^−5^ was applied. Additionally, protein sequences such as RdRp (RNA-dependent RNA polymerase), Rep (replication-associated protein), and NS1 (non-structural protein) were downloaded from the RefSeq database. These sequences were used to compare overlapping groups with lengths >1,500 bp. Taxonomic identification was performed using the rma2info program embedded in MEGAN6 (Gautam et al., 2022). The predicted open reading frames (ORFs) were forecasted using Geneious Prime (Kearse et al., 2012) and were compared with relevant viruses. The annotation of these ORFs relied on comparisons with the Conserved Domain Database (CDD). Using MEGAN6 (Huson et al., 2007), a standardization and comparison of the composition analyses for three libraries was conducted, and the results on viral community structure were described using the R v4.2.1 packages pheatmap.

### 2.3 Phylogenetic analysis

To infer phylogenetic relationships, nucleotide and protein sequences of reference strains from various hosts belonging to the respective viruses were downloaded from the NCBI GenBank database. Relevant nucleotide and protein sequences were aligned using the alignment program implemented in CLC Genomics 10.0 (https://digitalinsights.qiagen.com), and further optimized using MUSCLE in MEGA-X v10.1.8 (Kumar et al., 2018) and the E-INS-I algorithm in MAFFT v7.3.1 (Kuraku et al., 2013). Protein sequences were used to construct a Bayesian inference tree with MrBayes v3.2 (Ronquist et al., 2012), running the Markov chain for up to one million generations with sampling every 50 generations, discarding the first 25% of the Markov Chain Monte Carlo (MCMC) samples as burn-in. A maximum likelihood tree was also constructed to confirm all Bayesian inference trees using MEGA-X v10.1.8 (Kumar et al., 2018). The nucleotide sequences were analyzed using Sequence Alignment Tool v1.2 (Muhire et al., 2014) to determine the color-coded distance matrix between the three Cyclovirus sequences we excavated and other members of the Cyclovirus genus.

### 2.4 Prediction of spatial structure

ColabFold (Mirdita et al., 2022) was utilized to predict the three-dimensional structures of the viral structural proteins identified in this study, while UCSF ChimeraX (Goddard et al., 2018) was employed for visualization and analysis.

## 3 Results

### 3.1 Overview of human blood virome

**Figure 1:**
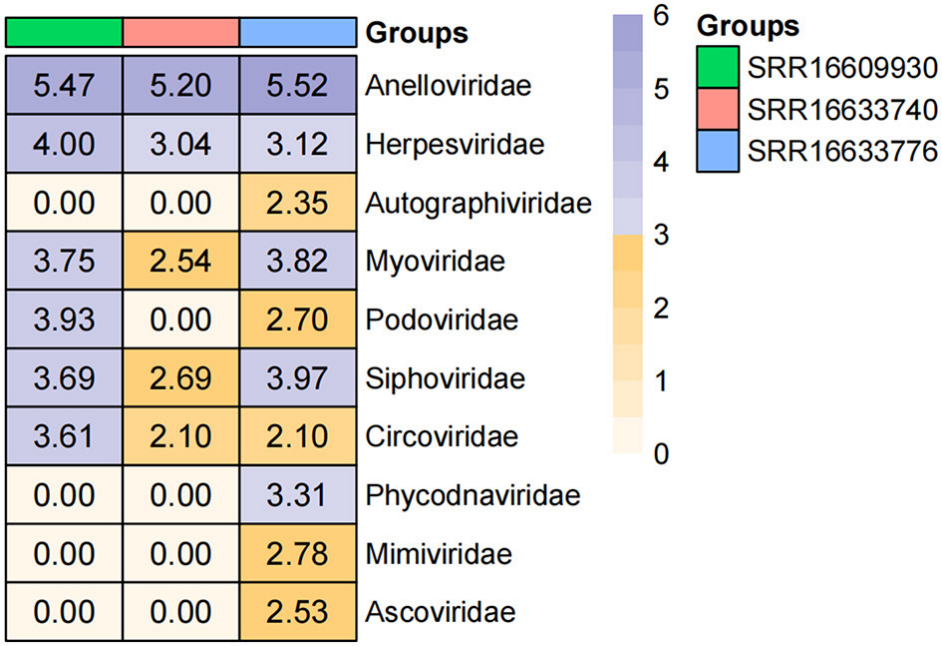
Statistical analysis of mining human blood virus communities. The heatmap was generated by transforming the read counts for each viral family in individual libraries into a log10 scale to normalize the data. The horizontal axis (x-axis) represents the different sample groups included in the study, while the vertical axis (y-axis) lists the different viral families detected in the samples. The color gradient in the heatmap indicates the relative abundance of each viral family, with darker colors representing higher abundance and lighter colors indicating lower abundance. Specifically, larger values of log-transformed read counts are depicted by darker shades, which correspond to more abundant viral families in the sample groups.

The three libraries generated a total of 30,006,599 raw reads on the Illumina HiSeq platform, resulting in 29,999,462 clean reads after quality control. A comparison of the clean reads with the nr database revealed 11,708,463 reads that matched the best viral protein, constituting 39.03% of the total clean reads. Ten viral families were detected, with the most abundant being *Anelloviridae* (790,177 reads, 93.34%), followed by *Siphoviridae* (148,280 reads, 1.75%), *Myoviridae* (125,450 reads, 1.48%), *Herpesviridae* (12,474 reads, 1.47%), *Podoviridae* (9,091 reads, 1.07%), *Circoviridae* (4,358 reads, 0.50%), *Phycodnaviridae* (2,064 reads, 0.24%), *Mimiviridae* (601 reads, 0.07%), *Ascoviridae* (340 reads, 0.04%), and *Autographiviridae* (223 reads, 0.02%). Additionally, there were 428,994 reads that could not be assigned to any taxonomic level (E-value > 10^−5^), which may represent potential novel viruses. Heatmap illustrates the differences in abundance among the three groups (SRR16609930, SRR16633740, and SRR16633776) across various viral families. The figure indicates that the abundance of *Anelloviridae* was consistently high in all three groups, exceeding 5, particularly in the SRR16609930 group, where it reached 5.47. Notably, *Anelloviridae* has been linked to immune modulation and inflammation, with higher viral loads observed in individuals with autoimmune conditions or chronic infections, such as HIV and hepatitis (Focosi et al., 2024). This could explain the observed high abundance across the groups, as immune status and chronic conditions are often correlated with elevated *Anelloviridae* levels. In contrast, the abundance of *Herpesviridae* was moderate in SRR16609930 and SRR16633740, but significantly increased in SRR16609930 to 4.00 (Figure 1). Previous research has demonstrated that *Herpesviridae* levels can fluctuate with host immune status, particularly during periods of immune suppression or reactivation. This suggests that the higher abundance of *Herpesviridae* in SRR16609930 may be related to specific immune conditions or viral reactivation in the host. Similarly, *Myoviridae* and *Podoviridae* exhibited inter-group variations, with SRR16633776 showing higher abundance of *Myoviridae* compared to the other groups. Furthermore, certain viral families, such as *Ascoviridae*, were undetected in SRR16609930 but were found in the SRR16633776 group at an abundance of 2.53. This observed variability in viral family abundance underscores the complex interplay between host immune factors and environmental conditions, and suggests that both biological and ecological factors contribute to the presence and diversity of viral families in these groups.

### 3.2 Identification of novel cycloviruses

**Figure 2:**
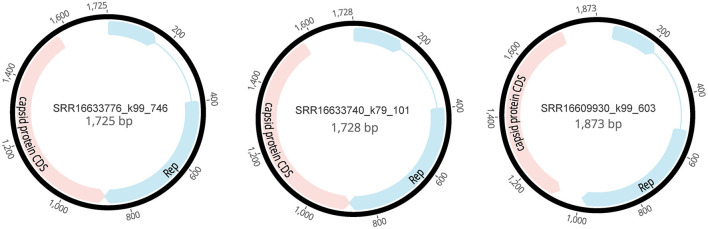
Genomic structures of three novel cycloviruses identified in the mined human blood genomic library.

In this study, the sequences of *Circoviridae* (4,358 reads) were subjected to de novo assembly using Geneious Prime v2019.0.4, successfully yielding three complete genomes associated with the family *Circoviridae*, designated as SRR16609930_k99_603 (1,873 bp), SRR16633740_k79_101 (1,728 bp), and SRR16633776_k99_746 (1,725 bp). Each of these complete genomes contains two open reading frames (ORFs), which encode the Rep and Capsid proteins (Rosario et al., 2017). The accuracy of these assemblies was validated through BLASTx comparison (Figure 2). This finding provides significant genomic data for the classification and functional studies of *Circoviridae*.

### 3.3 Distance matrix analysis of genes

**Figure 3:**
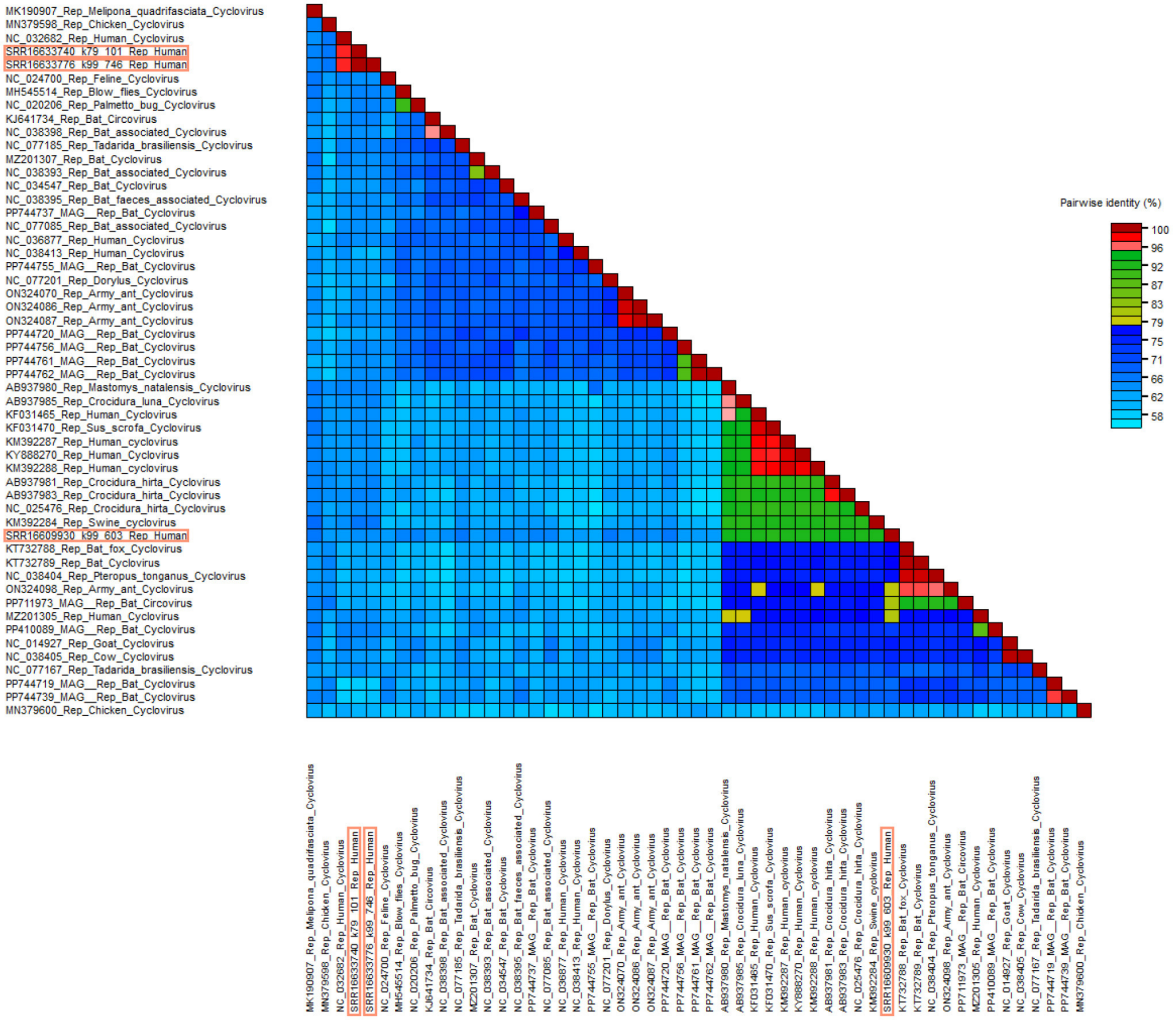
Distance matrix analysis of *Circoviridae*. Pairwise sequence comparison produced with Rep proteins nucleotide sequences for cycloviruses. Sequences framed in orange are new sequences discovered in this study.

According to the distance matrix data, there is a high similarity between SRR16633740_k79_101 and SRR16633776_k99, both exhibiting the highest similarity score of 97.05% with NC_032682 (Figure 3). Additionally, the similarity score between AB937980 and SRR16609930_k99_603 is 92.52%. These results indicate a relatively high similarity between the *Circoviridae* viral sequences of Mastomys natalensis and those of human *Circoviridae*. Notably, the similarity scores for the Rep protein gene sequences of other known Circoviridae viruses from the African region are all above 90% when compared to SRR16609930_k99_603. These data suggest that this viral group may primarily circulate between animals and humans in Africa.

### 3.4 Phylogenetic analysis of *Circoviridae*

**Figure 4:**
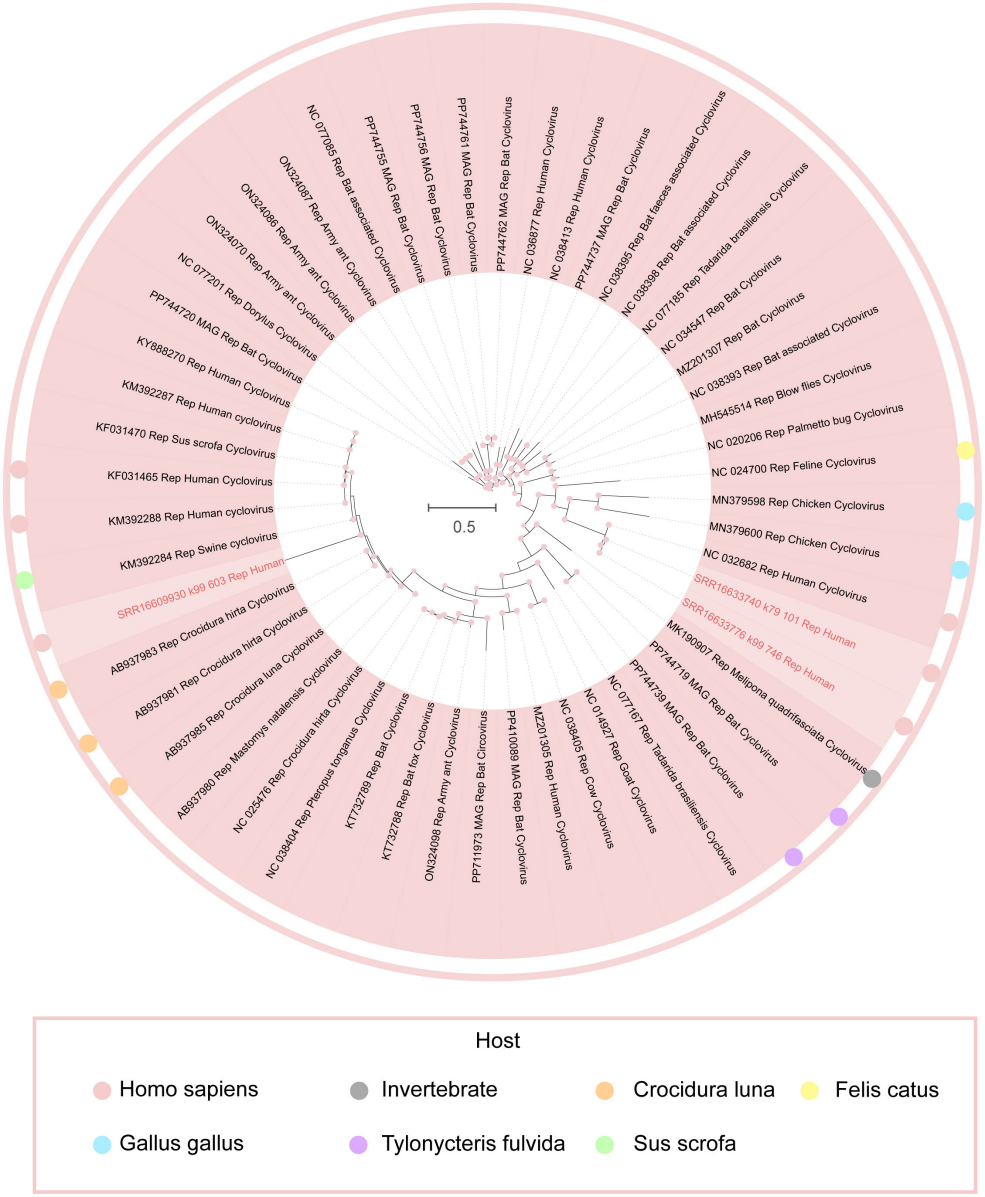
Phylogenetic relationship of *Circoviridae*. Phylogenetic analysis tree was constructed based on the amino acid sequences of Rep proteins for cycloviruses. Each of the different colored dots in the legend represents a host organism, and the red dots represent this study. Each bar represents an amino acid substitution at each site. Nodes colored red indicate a support value >75%.

In this study, a phylogenetic tree was constructed based on the Rep amino acid sequences of the newly discovered cycloviruses to elucidate their relationships with known viral families (Varsani et al., 2024). By comparing the Rep sequences of these newly identified cycloviruses with sequences from established families, we were able to infer their evolutionary relationships and position within the current viral taxonomy. Coupled with BLASTx results from the NCBI database, we successfully classified these three newly recognized complete cycloviruses under the genus Cyclovirus within the *Circoviridae* family (Figure 4).

Notably, phylogenetic analysis revealed that SRR16633740_k79_101 and SRR16633776_k99_746 form a distinct cluster with the known NC_032682. The BLASTx comparison indicated similarity scores of 93.24% and 93.67%, respectively, with NC_032682, derived from cerebrospinal fluid in India, while similarity to other known viral family sequences was below 65%, suggesting the potential formation of a new subfamily. Additionally, SRR16609930_k99_603 displayed a similarity of 91.77% with AB937980, obtained from the feces of *Mastomys natalensis* in Zambia, and both showed ≥87% similarity to various viral sequences from African animals and humans. These data support the hypothesis that this viral group predominantly circulates between animals and humans in Africa, with the phylogenetic clustering of these newly discovered cycloviruses in close relation to known African viral species suggesting potential zoonotic spillover.

The phylogenetic distances observed between these cycloviruses and other related viral sequences suggest that while these new viruses share a recent common ancestor with known species, their distinct clustering also points to a possible divergence driven by ecological and evolutionary pressures, such as host adaptation and environmental factors. The high similarity to viral sequences found in both humans and animals within Africa indicates that the virus may have a broad host range, and the observed phylogenetic patterns may reflect ongoing interspecies transmission. This further supports the idea that these cycloviruses could be circulating between human and animal populations, potentially through direct contact or shared ecological niches, and may be subject to selective pressures that favor their persistence across diverse host species.

These findings underscore the zoonotic potential of these cycloviruses and the need to investigate potential transmission routes, such as spillover events from animal to human populations. The clustering of viral sequences from Africa also raises questions about the role of regional ecological factors—such as wildlife trade, habitat encroachment, or changes in animal migration patterns—in facilitating cross-species transmission. Further studies examining the host range, transmission dynamics, and evolutionary pressures acting on these cycloviruses are essential to understanding their ecological and public health significance.

### 3.5 Rep protein spatial structure prediction

**Figure 5:**
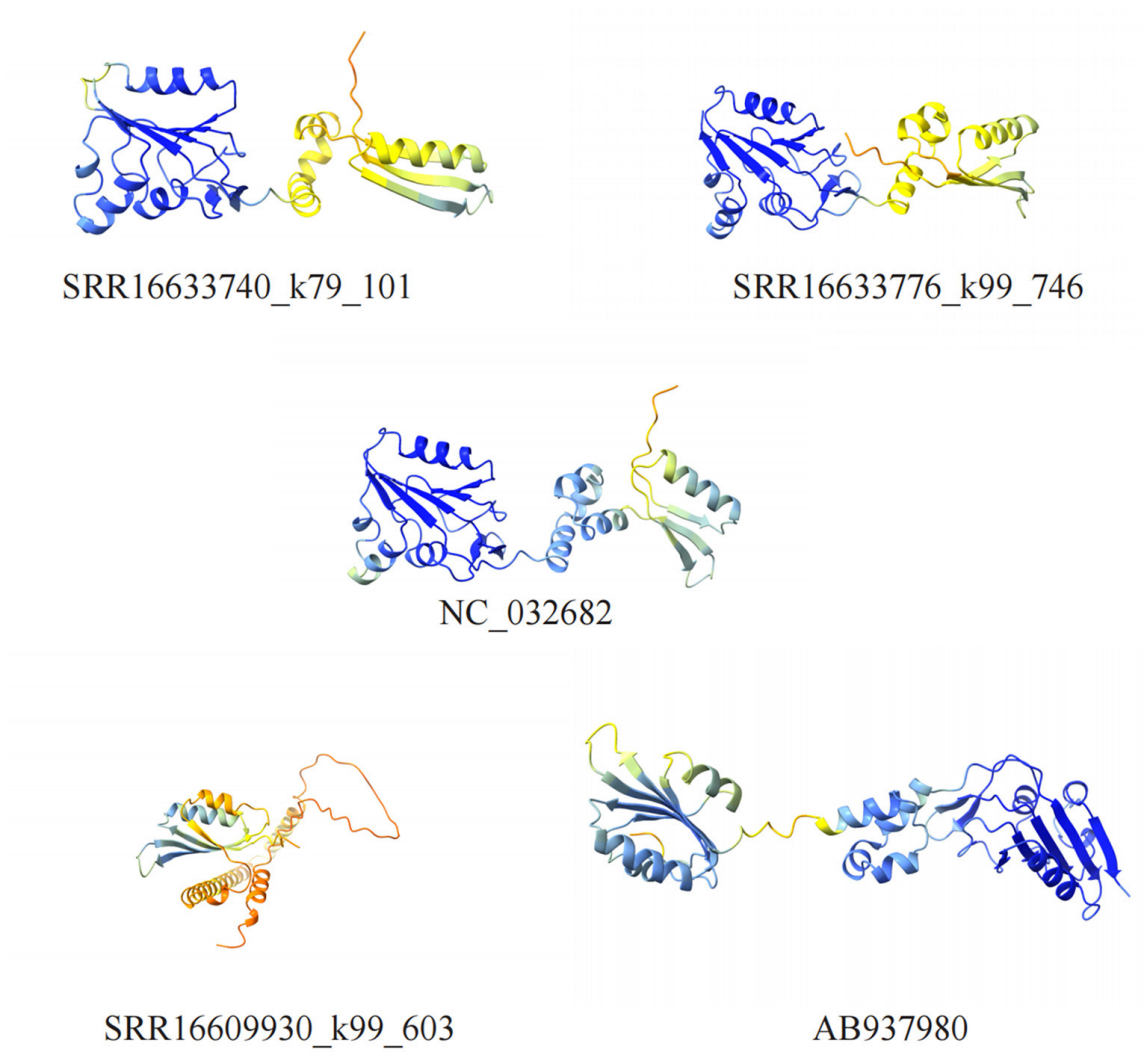
Visualization of cycloviruses Rep protein structure models. Five different structural models of proteins are shown, identified by different numbers, which contain α-helices and β-sheets regions.

The Rep protein of the *Circoviridae* family plays a crucial role in the viral lifecycle, with primary functions including promoting the replication of viral DNA as a vital enzyme, regulating viral RNA transcription to enhance protein synthesis, participating in RNA splicing to generate mature mRNA, and influencing host cell metabolism and immune responses (Luo et al., 2018). To predict and compare the spatial structures of the Rep proteins identified in this study with known sequences, we downloaded NC_032682 and AB937980 from the GenBank database and utilized the ColabFold tool for spatial structure predictions (Figure 5).

The structural similarity between SRR16633740_k79_101, SRR16633776_k99_746, and NC_032682 is striking, indicating not only evolutionary conservation but also functional similarity. The overall conformations of these Rep proteins are highly similar, with well-aligned α-helices and β-sheets, suggesting that these proteins likely perform similar roles in viral replication and host interaction. This conservation points to the stability of the structural elements critical for DNA replication, as well as for the modulation of host immune responses. Given the critical role of Rep in these processes, such structural consistency is likely necessary for maintaining viral fitness and the virus's ability to evade immune detection and modulate host cell activities.

However, SRR16609930_k99_603 and AB937980, despite showing a sequence similarity of 91.77%, exhibit notable structural differences. These differences are most evident in the spatial arrangement of certain key α-helices and β-sheets, which appear to be displaced or altered compared to NC_032682. Specifically, mutations in a few critical amino acids likely lead to shifts in these secondary structure elements, resulting in a reorganization of the protein's overall tertiary structure. These changes could have profound implications for the Rep protein's function. For instance, the altered structural configuration may affect the protein's ability to interact with host replication machinery, potentially reducing the efficiency of viral DNA replication. Additionally, structural differences in the Rep protein could also impact the virus's ability to infect different host species. If these mutations alter how the Rep protein binds to host cell receptors or interact with host immune components, it could enable the virus to adapt to new host environments, thus facilitating zoonotic spillover or cross-species transmission.

## 4 Discussion

In recent years, the discovery and characterization of cycloviruses in various environments have increased significantly due to advancements in next-generation sequencing technology (Jiang et al., 2023). While some of these newly identified viruses are associated with diseases, most pose limited threats to humans or animals (Delwart and Li, 2012). However, their potential pathogenicity cannot be overlooked, making it essential to investigate the impacts of these viruses in depth for the development of effective prevention and treatment strategies (Li et al., 2010).

This study systematically explores the classification, functions, and interactions of cycloviruses with their hosts. Through quality control and alignment of raw sequencing data generated on the Illumina HiSeq platform, we identified ten viral families, with *Anelloviridae* demonstrating significant abundance, indicating its dominance in environmental samples (Liu et al., 2012). This disparity in abundance may be related to the ecological environment of the sample source, the immune status of the host, or biodiversity. The *de novo* assembly analysis of cycloviruses successfully revealed three new genomes, each encoding Rep, and Capsid proteins, laying the groundwork for further viral research.

Distance matrix analysis showed that these cycloviruses viral sequences exhibit high similarity with viruses from humans and other regions of Africa, suggesting a potential for transmission between animals and humans. This finding provides critical insights into the evolutionary relationships of cycloviruses and establishes a foundation for assessing their potential public health risks.

Phylogenetic analysis indicates that the newly identified cycloviruses form a distinct evolutionary cluster with the known genus cycloviruses, further confirming their taxonomic status and potentially leading to the establishment of new subfamilies. This revelation of evolutionary relationships enhances our understanding of the origins and transmission pathways of these viruses, particularly in the biodiversity-rich African region. Interestingly, SRR16609930_k99_603 shows high similarity to various cycloviruses derived from African animals and humans, exhibiting a clear distance in the phylogenetic tree. This suggests the possible existence of previously undiscovered cycloviruses in Africa. The high degree of genetic similarity observed among these viruses may point to a common ancestor, raising the possibility of cross-species transmission or zoonotic spillover events. A case in point is the identification of a novel human circovirus (HCirV-1) in an immunocompromised 66-year-old woman with self-limiting hepatitis, where the virus persisted in her blood, stool, and urine over a long period (Hamelin et al., 2024). This case highlights the potential for circoviruses to persist in human hosts, particularly under conditions of immune suppression, and underscores the importance of investigating the broader implications of cyclovirus transmission between animals and humans.

However, it is important to acknowledge the limitations of inferring transmission pathways solely from phylogenetic data. While the clustering of viral sequences does suggest evolutionary relatedness and potential zoonotic transmission, phylogenetic trees alone cannot definitively establish transmission routes or host adaptation mechanisms. Further research, including epidemiological studies and direct host-virus interaction studies, would be required to confirm the exact pathways of transmission and to understand the factors driving host adaptation and spillover events.

Rep proteins are key enzymes involved in viral DNA unwinding and replication, and their tertiary structure is crucial for the proper execution of their functions (Luo et al., 2018). Amino acid mutations can significantly impact their folding, stability, and interactions with host proteins, thereby affecting viral replication efficiency and immune evasion. A comparative analysis of three Rep protein sequences (SRR16609930_k99_603, SRR16633740_k79_101, and SRR16633776_k99_746) reveals how amino acid changes influence the structure and function of Rep proteins.

In sequence SRR16609930_k99_603, the MANNSTVR region differs markedly from sequences SRR16633740_k79_101 and SRR16633776_k99_746, with mutations such as Ala → Glu, Ser → Lys, Thr → Val, and Pro → Gly. These changes can result in local charge alterations, hydrophobicity shifts, and secondary structure disruptions, all of which can destabilize the overall protein conformation. For example, the substitution of Ala with Glu could destabilize the local structure by disrupting hydrogen bonds or ionic interactions, while the Pro-to-Gly change may lead to the loss of stable helical structure, thereby affecting unwinding activity and DNA binding affinity.

Moreover, amino acid substitutions may alter the interactions of Rep protein with both DNA and host factors. For instance, the Ser → Lys mutation could enhance the Rep protein's DNA-binding affinity, thereby improving its helicase activity. Conversely, the Pro → Gly mutation might destabilize the protein's folding, reducing its interaction with DNA and lowering replication efficiency. Rep proteins also interact with various host proteins, such as DNA polymerases and transcription factors, and changes in amino acid residues may disrupt these interactions, affecting viral replication and transcription.

In conclusion, amino acid mutations in Rep proteins influence their structure and function through multiple mechanisms, ultimately impacting viral replication efficiency and immune evasion capabilities. These findings not only shed light on viral adaptive evolution but also provide potential targets for antiviral strategy development.

This study confirms the phylogenetic position of cycloviruses and emphasizes the need for further research to fully understand their ecological roles and potential public health risks. While the current findings, based on datasets from specific African regions, provide valuable insights into cyclovirus diversity in biodiversity-rich areas, their limited geographic scope highlights the need for broader studies. Future research should focus on targeted epidemiological studies in diverse regions, including both high-risk areas like Africa and regions with varying environmental conditions and public health infrastructures. These studies will be essential for tracking cyclovirus prevalence in wildlife, livestock, and humans, identifying transmission hotspots, and assessing environmental factors influencing spillover events. Expanding research to include different populations will help determine regional variations in host associations, pathogenicity, and epidemiology, improving our understanding of their global public health implications. In addition, *in vitro* pathogenicity studies are needed to explore cyclovirus replication, cytotoxicity, and immune interactions, particularly in immunocompromised hosts. Longitudinal monitoring of cyclovirus prevalence in human and animal populations will be crucial for early detection of emerging zoonotic threats and for strengthening surveillance systems. By expanding the geographic scope and incorporating diverse populations, these efforts will enhance our understanding of cyclovirus biology and inform better strategies for managing their potential impact on human and animal health.

## Data Availability

The novel cycloviruses sequences obtained in this study have been submitted to the GenBank database. The accession numbers are BK068984, BK068985, BK068986.
